# Primary Pap Smear Cytological Diagnosis of ASC-US: HR-HPV Genotyping and Follow-Up of HPV-Positive Patients

**DOI:** 10.3390/cancers18183022

**Published:** 2026-09-17

**Authors:** Tiziana Pisani, Maria Cenci, Ettore Domenico Capoluongo

**Affiliations:** 1 Unità Operativa Complessa di Patologia Clinica, Azienda Ospedaliera San Giovanni-Addolorata, 00184 Rome, Italy; tpisani@hsangiovanni.roma.it (T.P.); mcenci@hsangiovanni.roma.it (M.C.); 2 Dipartimento di Medicina Molecolare e Biotecnologie Mediche, Università Federico II, 80138 Napoli, Italy

**Keywords:** atypical squamous cells of undetermined significance (ASC-US), high-risk human papillomavirus (HR-HPV), cervical cancer screening, extended HPV genotyping, cervical cancer

## Abstract

ASC-US refers to cytological abnormalities suggestive of squamous intraepithelial lesions (SILs) that are qualitatively or quantitatively insufficient for a definitive diagnosis. In addition to HPV infection, which is the etiological agent of preneoplastic and neoplastic cervical lesions, several other conditions may induce cytological changes classified as ASC-US, including inflammation, atrophy with degeneration, hormonal effects, and technical artifacts. The aim of this study was to evaluate the incidence of primary Pap tests diagnosed as ASC-US that also tested positive for HR-HPV genotypes. In this retrospective study, 6181 primary Pap tests collected between 21 July 2021 and 9 January 2024 from the Roma 2 Local Health District were reviewed. ThinPrep containers (Hologic) were used for liquid-based cytology, allowing both cytological examination and molecular testing for HR-HPV detection. The Anyplex™ II HR HPV Detection assay (Seegene) was used for the identification, by real-time PCR, of 14 HR-HPV genotypes (16, 18, 31, 33, 35, 39, 45, 51, 52, 56, 58, 59, 66, and 68). Among the 6181 Pap tests analyzed, 202 cases (3.3%) were diagnosed as ASC-US and subsequently underwent HR-HPV molecular testing. Of these, 107 cases (53.0%) were negative, while 95 (47.0%) were positive for HR-HPV. In HPV-positive samples, single-genotype infections were detected in 65 cases (68.4%), while multiple genotypes were identified in 30 cases (31.6%). A total of 135 HR-HPV genotypes were detected. HPV 16 was identified in nine cases (6.7%), HPV 18 in six (4.4%), HPV 31 in nine (6.7%), HPV 33 in five (3.7%), HPV 35 in two (1.5%), HPV 39 in 10 (7.4%), HPV 45 in four (3.0%), HPV 51 in 20 (14.8%), HPV 52 in seven (5.2%), HPV 56 in 15 (11.1%), HPV 58 in eight (5.9%), HPV 59 in 17 (12.6%), HPV 66 in 12 (8.9%), and HPV 68 in 11 cases (8.1%). Our findings indicate that approximately half of ASC-US diagnoses are associated with HR-HPV infection. Interestingly, the detected viral genotypes mainly belonged to less common HR-HPV strains. HR-HPV testing following an ASC-US cytological diagnosis is essential for colposcopy triage, while extended genotyping enables the monitoring of genotype persistence over time, a key factor in the development of cervical dysplastic and neoplastic lesions.

## Simple Summary

The interpretation of cervicovaginal cytology samples can sometimes present difficulties primarily due to the presence of morphological alterations that are not open to unequivocal interpretation. Such samples are reported as atypical squamous cells of undetermined significance (ASC-US) and selected for high-risk human papillomavirus (HR-HPV) testing. In this study, we aimed to evaluate the incidence of samples testing positive for high-risk viral genotypes, identify which genotypes were most frequently involved, and assess the grade of dysplasia found in the subsequent biopsy.

## 1. Introduction

Human papillomavirus (HPV) screening strategies may vary across different countries. However, following a health technology assessment (HTA) evaluation, Italian cervical screening programs progressively replaced cytology-based screening with primary HPV DNA testing, supported by evidence from European clinical trials [1] and initial local pilot experiences [2]. The implementation was deliberately gradual to facilitate the transition from three- to five-year screening intervals without causing discontinuity in the workload of sample collection services, molecular and cytology laboratories, and colposcopy clinics. The Italian screening algorithm [2] adopts one of the most conservative approaches recommended by European guidelines [3]: HPV-positive women undergo cytological triage, with immediate colposcopy for those showing atypical squamous cells of undetermined significance (ASC-US) or worse lesions, while women with negative cytology are recalled for HPV testing after one year.

Approximately 3–10% of women are diagnosed with ASC-US [4].

Among cytological abnormalities, ASC-US represents a challenging category due to its uncertain clinical significance. Histopathological evaluation of ASC-US cases may reveal a broad spectrum of findings, including reactive/inflammatory alterations, cervical intraepithelial neoplasia (CIN), or, less frequently, cervical carcinoma. Furthermore, according to the Bethesda System for reporting cervical cytology, ASC-US represents a category of cervical epithelial abnormalities suggestive of squamous intraepithelial lesions (SILs), although qualitatively and quantitatively insufficient for a definitive SIL diagnosis [5].

Several non-neoplastic conditions may produce cytological changes leading to an ASC-US diagnosis, including inflammation, atrophy with degeneration, hormonal effects, and technical artifacts [5,6]. Since persistent infection with HPV is recognized as the main etiological factor for preneoplastic and neoplastic cervical lesions, HPV DNA testing represents a highly sensitive and specific tool for distinguishing reactive cytological changes from dysplastic lesions [7,8].

Some recent studies focused on the performance of different triage strategies for women presenting with ASC-US in the pre- and post-HPV vaccination era, providing insights into the optimization of cervical cancer prevention strategies, particularly in resource-constrained healthcare systems [4], to reduce the risk of overtreatment [9]. Moreover, the role of genotyping is still under debate in terms of cost-effectiveness, particularly for the management of ASC-US lesions [2].

Nevertheless, more than 400 HPV genotypes have been identified; however, only 14 are currently classified as high-risk (HR) genotypes associated with cervical carcinogenesis [10]. Based on carcinogenic potential, HPV 16 and 18 are considered the highest-risk genotypes, whereas HPV 31, 33, 35, 45, 52, and 58 are associated with intermediate risk, and HPV 39, 51, 56, 59, 66, and 68 with lower oncogenic risk [11].

Current European guidelines for cervical cancer screening recommend the implementation of clinically validated high-risk HPV DNA assays as the primary screening modality in women over 30 years of age. To ensure analytical quality, reproducibility, and efficiency, testing should be centralized in qualified laboratories and performed using automated platforms capable of integrating all phases of the workflow [12].

The Lazio Region cervical cancer (CC) screening program recommends Pap testing every three years for women aged 25–29 years. Moreover, women aged 30–64 years undergo primary HPV-DNA testing at 5-year intervals. In cases of ASC-US cytology, HR-HPV DNA testing is performed as a triage test [13,14]. Patients testing positive for HR-HPV are referred for second-level investigations, including colposcopy, possible biopsy, and subsequent follow-up or surgical treatment when indicated [15].

The aim of the present study was to evaluate (a) the incidence of HR-HPV positivity among primary Pap tests diagnosed as ASC-US; (b) the HR-HPV genotype distribution among ASC-US cases in the Lazio region; and (c) how the ASC-US-associated genotypes correlate with patients’ outcomes, also in relation to the vaccination status of our patient cohort.

## 2. Materials and Methods

The present study is based on data collected through the cervical cancer screening program implemented by the Regional Health Service of Lazio, Italy. According to the regional screening protocol, cervical cytology (Pap test) is performed every three years in women aged 25–29 years, whereas high-risk human papillomavirus (HR-HPV) testing is performed every five years in women aged 30–64 years. The Clinical Pathology Laboratory of San Giovanni Addolorata Hospital in Rome is one of the two regional laboratories designated for HPV-based cervical cancer screening. Our laboratory receives and processes cervical samples collected from four areas of the Lazio Region: Frosinone, ASL Roma 2, ASL Roma 6, and Latina.

Cervical cytobrush specimens are collected at the four healthcare facilities (Frosinone, ASL Roma 2, ASL Roma 6, and Latina) participating in the regional screening program and subsequently transported to the laboratory in ThinPrep^®^ PreservCyt^®^ Solution. Liquid-based cytology was performed using ThinPrep^®^ PreservCyt Solution (Hologic, Marlborough, MA, USA), which allows both preparation of thin-layer slides for microscopic examination and molecular testing for HR-HPV detection. The Anyplex™ II HR HPV Detection assay (Seegene, Seoul, Republic of Korea) was used to detect 14 HR-HPV genotypes (16, 18, 31, 33, 35, 39, 45, 51, 52, 56, 58, 59, 66, and 68) by real-time PCR. Statistical analysis was performed using the SG STATS platform (Seegene, Seoul, Republic of Korea) [13,14].

The screening results are entered into the regional SIPSO software platform (version 2.0), which enables the transmission of screening results to the referring healthcare facilities and ensures appropriate, patient-specific follow-up. Samples testing positive by molecular HPV screening are subsequently referred to the Pathology Unit for cytopathological evaluation and reporting. Implicit consent is obtained from all women participating in the screening program at the time of the gynecological examination performed at the outpatient facilities located across the four territorial areas described above. In fact, regional screening is included among the essential levels of care (LEA) provided for by the Italian National Health Service and therefore does not require specific informed consent (as provided for by Italian national legislation). Once a woman, after receiving an invitation letter to attend the gynecological clinic closest to her place of residence, agrees to participate in the screening program, she is enrolled within the national and regional cervical cancer prevention policies. The SIPSO regional platform contains comprehensive data on all women undergoing cervical cancer screening, including information on HPV vaccination status (when available), and enables statistical analyses to be performed on aggregated, anonymized data. These analyses provide the epidemiological information required to monitor the prevalence and distribution of HPV genotypes in the Lazio population and to support the regional department in optimizing screening strategies, including the intensification of screening activities and the appropriate scheduling or modification of follow-up for women with positive screening results. We therefore used the SIPSO software to perform our retrospective study. Grade 1 or Grade 2 classifications were used: colposcopic examination included assessment of the cervical transformation zone according to the International Federation for Cervical Pathology and Colposcopy (IFCPC) classification: G1, in which the transformation zone is completely visible and entirely ectocervical, and G2, in which the transformation zone is completely visible but has an endocervical component. CIN1, CIN2, and CIN3 histopathological categories describe the grade of cervical intraepithelial neoplasia (CIN), namely precancerous changes affecting the cervical epithelium.

Statistical analysis: Odds ratios (ORs) with 95% confidence intervals (CIs) were calculated to assess the association between vaccination status and histological outcome, categorized as CIN1 versus high-grade lesions (≥CIN2). ORs were also calculated to evaluate the association between individual high-risk HPV genotypes and the presence of ≥CIN2 lesions. Given the small sample size of the vaccinated group (*n* = 7), Fisher’s exact test was used for categorical variables, while the Mann–Whitney U test was used to compare age.

A total of 6181 primary Pap tests collected between 21 July 2021 and 9 January 2024 were reviewed in order to identify cases diagnosed as ASC-US. Subsequently, the clinical records of patients who tested positive for HR-HPV were analyzed.

## 3. Results

**Figure 1:**
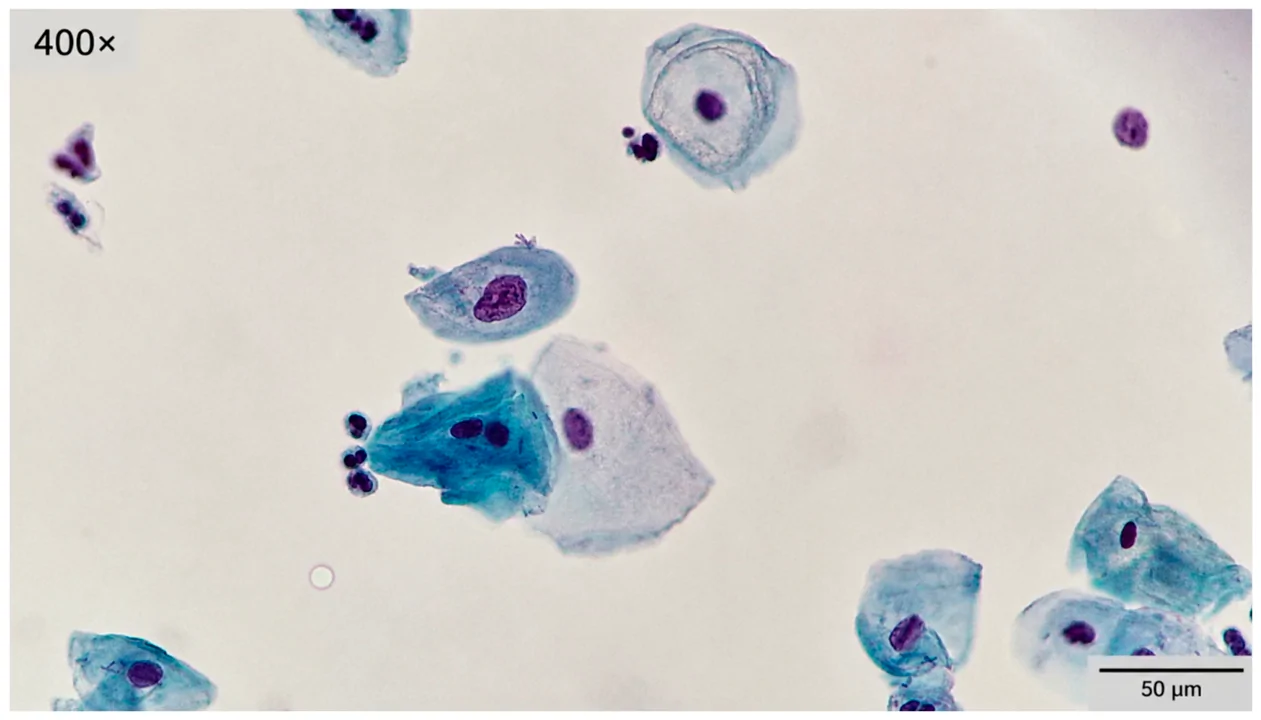
ASC-US cells from a ThinPrep slide. ASC-US cells showed a slight increase in the nuclear/cytoplasmic ratio, hyperchromasia, mild nuclear irregularity, and slight perinuclear halos.

**Figure 2:**
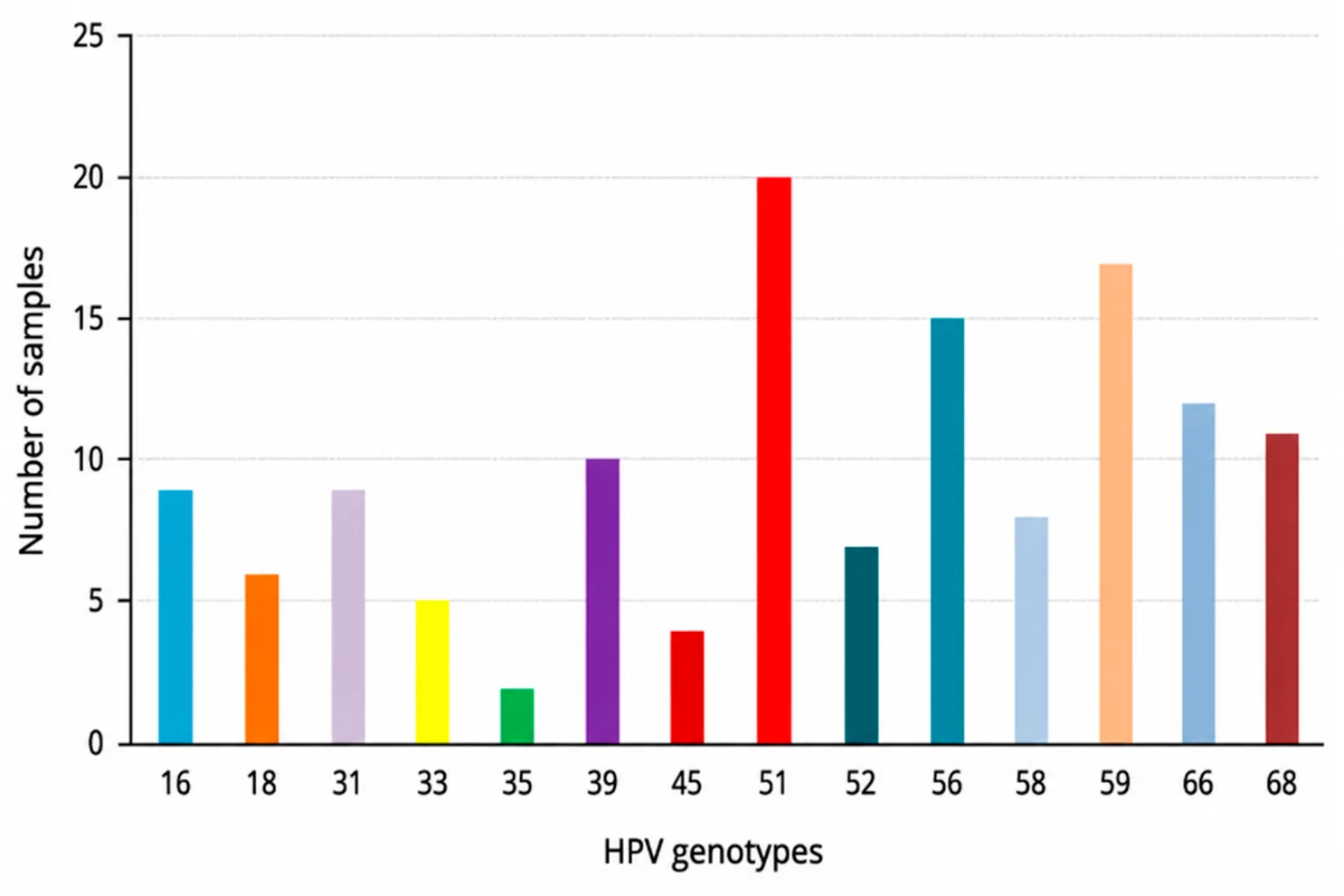
HR HPV genotypes detected in the 202 ASC-US cases (*n* = 202).

Among the 6181 Pap tests analyzed, 202 cases (3.3%) were diagnosed as ASC-US (Figure 1) and subsequently underwent HR-HPV molecular testing (Figure 2). Of these, 107 cases (53.0%) were negative for HR-HPV, whereas 95 cases (47.0%) tested positive.

Among HR-HPV-positive samples, a single genotype was identified in 65 cases (68.4%), while multiple HR-HPV genotypes were detected in 30 cases (31.6%). A total of 135 HR-HPV genotypes were identified. HPV 16 was detected in nine cases (6.7%), HPV 18 in six (4.4%), HPV 31 in nine (6.7%), HPV 33 in five (3.7%), HPV 35 in two (1.5%), HPV 39 in 10 (7.4%), HPV 45 in four (3.0%), HPV 51 in 20 (14.8%), HPV 52 in seven (5.2%), HPV 56 in 15 (11.1%), HPV 58 in eight (5.9%), HPV 59 in 17 (12.6%), HPV 66 in 12 (8.9%), and HPV 68 in 11 cases (8.1%) (Figure 2).

All HR-HPV-positive patients were referred for colposcopy. Two patients did not attend the examination. Colposcopy revealed abnormalities within the transformation zone in 47 cases (50.5%), whereas no alterations were detected in 46 cases (49.5%). Among patients without detectable colposcopy abnormalities, only four (8.7%) were positive for HPV 16 or 18 (one case HPV 16 + ve and three cases HPV 18 + ve).

Patients with colposcopically detectable abnormalities underwent targeted biopsy. Histological examination was negative in five cases (10.6%), while low-grade cervical intraepithelial neoplasia CIN grade 1 (CIN1) was diagnosed in 33 cases (70.2%). Moreover, high-grade cervical intraepithelial neoplasia CIN2 was found in five cases (10.6%), and high-grade cervical intraepithelial neoplasia CIN3 was found in two cases (4.2%) (Table 1). Patients with negative histology or low-grade lesions were scheduled for follow-up after one year, whereas patients diagnosed with high-grade cervical intraepithelial neoplasia CIN2/3 underwent conization.

Among the CIN-positive women, 15/40 (37.5%) had infections with ≥2 HPV genotypes. Multiple HPV genotype infections were less frequently observed among vaccinated women than among unvaccinated ones (14.3% vs. 42.4%); however, this difference did not reach statistical significance (Fisher’s exact test, *p* = 0.224). Among the CIN1 lesions, the distribution of HPV genotypes showed a predominance of several high-risk genotypes. HPV59 was the most frequently detected genotype (*N* = 8), followed by HPV39 and HPV51 (*N* = 7, respectively). HPV56 was identified in five cases, while HPV16, HPV66, HPV31, and HPV68 were each detected in four cases. Among the CIN2 lesions, the HPV genotype distribution was similarly heterogeneous, although the limited number of cases (*n* = 5) precludes any robust assessment of genotype-specific associations. HPV52 was the most frequently detected genotype (2/5 cases), while HPV18, HPV66, HPV33, HPV56, HPV16, and HPV31 were each detected in 1/5 cases. Finally, in the CIN3 group (*n* = 2), HPV16 was detected in both cases (2/2, 100%), whereas HPV51 was detected in one of the two cases (1/2, 50%). As reported in Table 2, no statistically significant differences were observed between vaccinated and unvaccinated women in terms of histological outcome, colposcopic findings, or age. CIN2–CIN3 lesions were observed in 28.6% of vaccinated women compared with 15.2% of unvaccinated women (Fisher’s exact test, *p* = 0.584). CIN3 lesions were detected exclusively among unvaccinated women (6.1% vs. 0%), although this difference was not statistically significant. Colposcopic findings were predominantly classified as G1 in both groups (93.9% vs. 85.7%; *p* = 0.448). Interestingly, HPV56 was more frequently detected among vaccinated women than among unvaccinated women (42.9% vs. 9.1%; OR = 7.48), approaching but not reaching statistical significance (*p* ≈ 0.06).

Vaccination data showed that 50 of the 202 women (24.7%) had received HPV vaccination. Most patients received the quadrivalent vaccine, while only one patient received the nonavalent one. Among vaccinated women, 30 (60%) were negative, while 20 (40%) were HR-HPV positive. None of the vaccinated patients tested positive for HPV 16 or 18. Only one vaccinated patient developed a high-grade squamous intraepithelial lesion (HSIL), associated with HPV genotype 56.

## 4. Discussion

In the mid-20th century, cervical cancer was among the leading malignancies affecting women worldwide [16,17,18]. However, the widespread implementation of effective prevention and screening strategies led to a sustained decline in both incidence and mortality across many high-income countries in North America and Europe, where cervical cancer is now considered a relatively uncommon disease [5,19].

To accelerate the elimination of cervical cancer as a public health problem, the WHO launched its global 90–70–90 strategy in 2020, targeting HPV vaccination of 90% of girls by age 15 years, screening of 70% of women with a high-performance test at ages 35 and 45 years, and treatment of 90% of women with precancerous cervical lesions or invasive cervical cancer [20]. Evidence from age–period–cohort analyses suggests that the implementation of cervical cancer screening programs in many countries prevented the resurgence in cervical cancer incidence that would otherwise have resulted from changes in sexual behavior [21]. However, the established etiological role of persistent HR-HPV infection in cervical carcinogenesis has made HR-HPV testing a cornerstone of modern screening. Previous studies demonstrated that HPV 16 and 18 confer the highest oncogenic risk, whereas HPV 31, 33, 35, 45, 52, and 58 are associated with intermediate risk, and HPV 39, 51, 56, 59, 66, and 68 with lower oncogenic risk [11,22]. Nevertheless, other cofactors may contribute to the persistence and progression of HPV-related dysplastic lesions [23,24]. The Lazio Region CC screening program recommends HR-HPV DNA testing every 5 years for women aged 30–65 years, whereas those aged 25–29 years undergo primary Pap testing every 3 years [13,25]. This strategy reflects the high prevalence but often transient nature of HPV infection in younger women, with spontaneous viral clearance occurring in approximately 60–90% of cases within 1–2 years [26,27].

The Pap smear remains an effective method for identifying cytological abnormalities associated with precancerous and malignant cervical lesions, where ASC-US encompasses a heterogeneous spectrum ranging from benign reactive changes to LSIL, HSIL, and, rarely, invasive squamous cell carcinoma [5,28]. ASC-US is the most common abnormal cytological interpretation, with reported frequencies ranging from 1.6% to 9.0%; however, expert consensus recommends that ASC-US diagnoses should not exceed 5% of all cytological reports [5,29].

The present study provides a detailed characterization of HR-HPV infection and genotype distribution among women diagnosed with ASC-US within a cervical cancer screening setting in Lazio, Italy. To the best of our knowledge, this represents the largest Italian cohort reported to date in the field of cervical cancer screening integrating cytological findings with molecular HPV testing and genotype characterization. The main findings can be summarized as follows: (i) ASC-US represented 3.3% of all Pap tests analyzed; (ii) HR-HPV infection was detected in approximately half of ASC-US cases (47.0%); (iii) single-genotype infections predominated, although multiple infections accounted for almost one-third of HR-HPV-positive women; (iv) the genotype distribution was heterogeneous and was characterized by a predominance of HPV51, HPV59, HPV56, HPV66, HPV68, and HPV39 rather than HPV16 or HPV18; (v) most histologically confirmed lesions were CIN1, whereas HPV16 was detected in both CIN3 cases; and (vi) vaccinated women showed a lower frequency of multiple HPV infections and no HPV16/18 infections, although the limited sample size prevented demonstration of statistically significant associations.

The frequency of ASC-US in our population was 3.3%, corresponding to 202 of 6181 Pap tests [11,30,31]. This value is within the range reported internationally and remains below the 5% threshold generally considered acceptable for ASC-US reporting in cervical cytology. This finding supports the overall quality and reproducibility of the cytological screening process in the participating population. Importantly, however, ASC-US represents a heterogeneous cytological category that may include both benign reactive alterations and HPV-associated lesions. Consequently, the identification of HR-HPV in 47.0% of ASC-US cases is clinically relevant because molecular testing provides an objective means of distinguishing women at increased risk of underlying cervical disease from those in whom the cytological atypia is more likely to be transient or reactive [29,30].

A particularly relevant finding of our study was the genotype distribution among HR-HPV-positive ASC-US samples. HPV51 was the most frequently detected genotype, followed by HPV59, HPV56, HPV66, HPV68, and HPV39, whereas HPV16 and HPV18 accounted for only a limited proportion of infections. This distribution differs from that reported in several European and North American screening cohorts, in which HPV16, HPV31, HPV52, HPV33, or HPV45 are more frequently represented among HPV-positive cervical abnormalities. Such differences may reflect geographical variation in HPV genotype prevalence, differences in vaccination coverage, population characteristics, assay methodology, and the clinical setting in which samples were collected. Therefore, our findings further support the concept that the distribution of HR-HPV genotypes in ASC-US cannot necessarily be extrapolated from data obtained in other populations [11,30,31,32,33].

The predominance of genotypes generally associated with lower oncogenic risk within the HR-HPV category is particularly interesting. These genotypes may induce subtle cytological changes that are more likely to be classified as ASC-US, whereas HPV16 and other highly oncogenic types may produce more overt lesions [11,29,30]. This hypothesis, however, requires confirmation in larger studies.

Most HR-HPV-positive ASC-US cases were associated with CIN1, although CIN2/3 lesions were also identified, supporting the value of HR-HPV testing for ASC-US triage: the HPV genotype distribution varied according to lesion grade. CIN1 lesions showed marked genotype heterogeneity, with HPV59 being the most frequently detected genotype, followed by HPV39 and HPV51, while HPV56, HPV16, HPV66, HPV31, and HPV68 were also represented. This broad distribution suggests that low-grade cervical lesions may be associated with a wide range of HR-HPV genotypes and that the presence of a genotype classified as lower oncogenic risk does not necessarily imply the absence of histologically detectable CIN. Importantly, however, CIN1 is frequently transient and has a substantially different biological significance from high-grade disease [28,29,31,32,33,34].

The CIN2 subgroup was also characterized by considerable genotype heterogeneity. HPV52 was detected in two of five cases, whereas HPV18, HPV66, HPV33, HPV56, HPV16, and HPV31 were each detected once. Because only five CIN2 cases were identified, these observations cannot support genotype-specific risk estimates. Nevertheless, the finding that CIN2 lesions were not restricted to HPV16 or HPV18 highlights the potential contribution of non-16/18 HR-HPV genotypes to clinically relevant cervical disease. This may be particularly important in populations undergoing progressive HPV vaccination, in which the relative contribution of non-vaccine or non-16/18 genotypes may become increasingly relevant.

In contrast, both CIN3 lesions in our cohort were associated with HPV16, with one case showing HPV16 alone and the other showing HPV16/HPV51 co-infection. Although the number of CIN3 cases was extremely small, this observation is consistent with the well-established biological association between HPV16 and high-grade cervical intraepithelial neoplasia. The apparent shift from a highly heterogeneous distribution of HPV genotypes in CIN1 and CIN2 to a predominant role of HPV16 in CIN3 is therefore particularly noteworthy.

The analysis of multiple HPV infections provides another potentially relevant observation. Multiple-genotype infections were identified in 31.6% of HR-HPV-positive ASC-US cases. Among women with CIN, 37.5% harbored two or more HPV genotypes. Multiple infections were less frequent among vaccinated women than among unvaccinated women (14.3% vs. 42.4%), although this difference did not reach statistical significance. The direction of this association is nevertheless biologically interesting and may be compatible with a reduction in infections involving vaccine-targeted genotypes and, consequently, a lower probability of concurrent infections. However, given the relatively small number of vaccinated women, this finding should not be interpreted as evidence of a protective effect against multiple infection. Larger prospective cohorts will be necessary to determine whether HPV vaccination modifies the frequency and composition of multiple HR-HPV infections.

The vaccination data are particularly relevant in the context of the ongoing transition toward HPV vaccination of increasingly large proportions of the population. In our cohort, 24.7% of women with ASC-US had previously received HPV vaccination, predominantly the quadrivalent vaccine. Among vaccinated women, 40% were HR-HPV positive; however, none of the vaccinated women carried HPV16 or HPV18. Only one vaccinated woman developed a high-grade lesion, associated with HPV56, a genotype not included in the quadrivalent vaccine. Although this observation is based on a very small number of events, it is consistent with the expected impact of vaccination on HPV16/18 infection. At the same time, the occurrence of HSIL associated with HPV56 illustrates that vaccination does not eliminate the possibility of infection with non-vaccine HR-HPV genotypes and supports the continued importance of cervical screening in vaccinated populations.

These findings have potentially important implications for future screening strategies. As vaccinated cohorts progressively enter the age range of organized cervical screening, the epidemiology of HPV-positive cytological abnormalities is expected to change. A reduction in HPV16/18-associated disease may result in a relative increase in the proportion of lesions associated with other HR-HPV genotypes. Consequently, extended HPV genotyping could become increasingly useful for risk stratification, particularly in women with persistent infection or abnormal cytology. Nevertheless, the clinical interpretation of individual genotypes should be integrated with age, vaccination status, persistence of infection, cytological findings, and previous screening history rather than being based solely on genotype detection.

Our data also have relevance for the management of ASC-US in organized screening programs. In the Lazio screening algorithm, HR-HPV-positive ASC-US cases are referred for colposcopy, whereas HPV-negative women can avoid immediate colposcopic assessment. The fact that approximately half of ASC-US cases in our cohort were HR-HPV negative illustrates the potential of HPV testing to reduce unnecessary diagnostic procedures. Conversely, the identification of CIN2/3 among HR-HPV-positive women confirms that ASC-US cannot be considered a uniformly low-risk cytological category. The combination of cytological triage and molecular HPV testing therefore remains a clinically effective approach for balancing sensitivity for clinically relevant lesions with the avoidance of unnecessary colposcopy.

Finally, we acknowledge that the retrospective study period partially overlapped with the COVID-19 pandemic, which may have affected screening participation, particularly during periods of restricted or suspended clinical services. However, following the resumption of routine screening activities, the number of samples analyzed returned to expected levels.

## 5. Conclusions

In conclusion, our study shows that HR-HPV-positive ASC-US in this Italian regional screening population is characterized by a heterogeneous genotype distribution, with a substantial contribution from HPV51, HPV59, HPV56, HPV66, HPV68, and HPV39. For HPV56 HPV genotype 56, no vaccine is currently available [35,36]. Most associated lesions were CIN1, whereas HPV16 was consistently detected in the two CIN3 cases, supporting its strong association with high-grade disease. The absence of HPV16/18 among vaccinated women and the lower frequency of multiple infections in vaccinated participants are encouraging findings, although they require confirmation in larger longitudinal cohorts. Overall, these results emphasize the value of HR-HPV testing in ASC-US triage and suggest that, as vaccinated cohorts become increasingly prevalent, extended HPV genotyping and vaccination status may become important components of individualized risk stratification. Future prospective studies with larger numbers of vaccinated women and high-grade lesions will be necessary to determine how vaccination modifies genotype distribution, persistence, and progression of HPV-associated cervical disease.

Extended genotyping also enables monitoring of genotype persistence, a crucial factor in cervical carcinogenesis [33,34], considering that in Europe, vaccination coverage ranges from 30.3% to 42.9%, depending on the country [34].

## Figures and Tables

**Table 1:** HR HPV genotype distribution among vaccinated and unvaccinated women with a histological diagnosis of cervical dysplasia.

Variable	U (*n* = 33)	V (*n* = 7)
CIN1	28 (84.8%)	5 (71.4%)
CIN2	3 (9.1%)	2 (28.6%)
CIN3	2 (6.1%)	0
Colposcopy G1—Transformation Zone Type 1	31 (93.9%)	6 (85.7%)
Colposcopy G2—Transformation Zone Type 2	2 (6.1%)	1 (14.3%)
HPV16	7	0
HPV18	3	0
HPV31	5	0
HPV33	1	1
HPV35	1	0
HPV39	7	0
HPV51	7	1
HPV52	3	1
HPV56	3	3
HPV59	6	2
HPV66	4	1
HPV68	4	0

V = Vaccinated; U = Unvaccinated.

**Table 2:** Comparisons between unvaccinated (U) and vaccinated women.

Variable	U (*n* = 33)	V (*n* = 7)	Statistical Test	*p*-Value
Age, median (years)	28	26	Mann–Whitney	0.092
CIN1	28 (84.8%)	5 (71.4%)	Fisher’s exact test	0.584
CIN2	3 (9.1%)	2 (28.6%)	Fisher’s exact test	— *
CIN3	2 (6.1%)	0 (0%)	Fisher’s exact test	1.000
CIN2–CIN3 (≥CIN2)	5 (15.2%)	2 (28.6%)	Fisher’s exact test	0.584
Colposcopy G1	31 (93.9%)	6 (85.7%)	Fisher’s exact test	0.448
Colposcopy G2	2 (6.1%)	1 (14.3%)	Fisher’s exact test	— *

* = not valuable. The odds ratio (OR) for a ≥CIN2 lesion in the vaccinated group compared with the unvaccinated group was approximately 2.24; however, the confidence interval would be very wide due to the small number of vaccinated subjects. Therefore, no statistically significant difference was observed.

## Data Availability

The data are automatically managed through the regional SIPSO platform, which is accessible exclusively to authorized and accredited healthcare professionals involved in the cervical cancer screening program. These data are used by authorized screening personnel to appropriately manage the samples requiring cytological examination and to ensure accurate and secure reporting of the screening results. Information regarding the vaccination status of individual patients is also available within the platform and may be particularly useful during the interpretation and reporting of HPV test results, as vaccination status can provide relevant clinical context for the appropriate evaluation of the findings. All data presented in this study were processed and reported in an aggregated and anonymized form, ensuring that individual patients cannot be identified.
